# An Essential Role of Mitochondrial α-Ketoglutarate Dehydrogenase E2 in the Basal Immune Response Against Bacterial Pathogens in Tomato

**DOI:** 10.3389/fpls.2020.579772

**Published:** 2020-10-30

**Authors:** Qiaomei Ma, Yaru Liu, Hanmo Fang, Ping Wang, Golam Jalal Ahammed, Wenshan Zai, Kai Shi

**Affiliations:** ^1^Department of Horticulture, Zhejiang University, Hangzhou, China; ^2^College of Forestry, Henan University of Science and Technology, Luoyang, China; ^3^Wenzhou Vocational College of Science & Technology, Wenzhou, China

**Keywords:** mitochondrion, α-ketoglutarate dehydrogenase E2, alternative oxidase, lipoylation, salicylic acid, *Solanum lycopersicum*, *Pst* DC3000, basal immunity

## Abstract

Plants intensely modulate respiration when pathogens attack, but the function of mitochondrial respiration-related genes in plant–bacteria interaction is largely unclear. Here, the functions of α-ketoglutarate dehydrogenase (α*-kGDH*) E2 subunit and alternative oxidase (*AOX*) were investigated in the interaction between tomato and the virulent bacterial pathogen *Pseudomonas syringae* pv. *tomato* DC3000 (*Pst*). *Pst* inoculation suppressed the transcript abundance of α*-kGDH E2*, but enhanced *AOX* expression and salicylic acid (SA) accumulation. Gene silencing and transient overexpression approaches revealed that plant susceptibility to *Pst* was significantly reduced by silencing α*-kGDH E2* in tomato, but increased by overexpressing α*-kGDH E2* in *Nicotiana benthamiana*, whereas silencing or overexpressing of *AOX1a* did not affect plant defense. Moreover, silencing octanoyltransferase (*LIP2*), engaged in the lipoylation of α-kGDH E2, significantly reduced disease susceptibility and hydrogen peroxide accumulation. Use of transgenic NahG tomato plants that cannot accumulate SA as well as the exogenous SA application experiment evidenced that α-kGDH E2 acts downstream of SA defense pathway. These results demonstrate tomato α*-kGDH E2* plays a negative role in plant basal defense against *Pst* in an AOX-independent pathway but was associated with lipoylation and SA defense pathways. The findings help to elucidate the mechanisms of mitochondria-involved plant basal immunity.

## Introduction

Plants growing in diverse environments are constantly challenged by a wide range of microbial pathogens and herbivorous insects that often result in growth inhibition and crop yield losses, leading to a significant risk to global agriculture. A plant mitochondrion is a membrane-bound organelle that occurred in the cytoplasm that primarily functions in the respiration to generate large quantities of energy in the form of ATP and carbon skeletons required for numerous plant biosynthetic processes, cellular maintenance, and active transport (Daloso et al., 2015). Many studies have suggested that leaf respiration is one of the most important metabolic processes in immune response (Colombatti et al., 2014). However, our understanding of the roles and mechanisms of mitochondria proteins during the immune response is largely unclear.

During evolution, plants have acquired a sophisticated immune system to mitigate the adverse effects of pathogen attack, i.e., effector-triggered immunity (ETI) and pattern-triggered immunity (PTI). ETI usually occurs in incompatible plant–pathogen interactions, in which plant Resistance (R) proteins detect the presence of avirulent pathogen effectors that are delivered inside plant cells (Jones and Dangl, 2006). In contrast, PTI often occurs in the compatible plant–pathogen interaction, which relies on the recognition of pathogen/damage-associated molecular patterns by cell surface pattern recognition receptors (Couto and Zipfel, 2016). For the susceptible host plants, PTI effectively repels most virulent pathogens, contributing to basal immunity (Couto and Zipfel, 2016).

Plant mitochondrial respiration is known to consist of glycolysis, the tricarboxylic acid (TCA) cycle, and mitochondrial electron transport chain (miETC). TCA cycle is a hub for cellular metabolism; it channels electrons from reduced substrates to the membrane-bound miETC for efficient energy conversion. A central enzyme of the TCA cycle is α-ketoglutarate dehydrogenase (α-kGDH), which catalyzes the oxidative decarboxylation reaction converting α-ketoglutarate, coenzyme A, and NAD^+^ to succinyl-CoA, CO_2_, and NADH, supplying the reducing equivalents for miETC in the form of NADH (Condori-Apfata et al., 2019). Prosthetic lipoyl groups are required for the function of α-kGDH, and the E2 subunit of α-kGDH protein was reported to be lipoylated by octanoyltransferase (LIP2) in *Arabidopsis* (Ewald et al., 2014). The plant miETC supporting oxidative phosphorylation branches at ubiquinone (UQ). The main pathway of respiration that flows from UQ through the usual cytochrome (Cyt) pathway operates via the four-electron reduction of O_2_ to H_2_O is the Cyt pathway, which leads to proton translocation at respiratory complexes I to IV, resulting in ATP production. In addition to this main pathway, plants also have a non-phosphorylating electron transport pathway that involves a protein called alternative oxidase (AOX) that catalyzes the direct oxidation of UQ and reduction of O_2_ to H_2_O, and bypass two-proton translocation at the sites of complexes III and IV, thus limiting ATP production (Vanlerberghe et al., 2020). Alternative respiration is widespread in plants, fungi, and some prokaryotes, but not in higher animals.

During plant–pathogen interactions, salicylic acid (SA) acts as a major defense hormone in plants, and it is required for the activation of the immune system and the development of systemic acquired resistance. Tissue SA levels increase in response to biotrophic and hemibiotrophic pathogens, and SA has been demonstrated to play a crucial role in plant defenses against a broad spectrum of pathogens, including viruses, oomycetes, fungi, and bacteria (López-Gresa et al., 2017; Qi et al., 2018). Intensive research efforts have been focused on the identification of SA-binding proteins (SABPs) and/or receptors. The non-expresser of pathogenesis-related gene 1 (*NPR1*) is a master regulator of SA signaling (Chen et al., 2019), which binds SA with a high affinity (Wu et al., 2012; Manohar et al., 2015). Two other SABPs, NPR3 and NPR4, were recently shown to act as negative regulators that fine tune SA signaling responses (Ding et al., 2018). The function of SA in plant defense has also been linked to mitochondrial metabolism or signaling (Poór, 2020). In the mitochondria, the E2 unit of α-kGDH protein was identified as SABPs in *Arabidopsis* and tomato (Tian et al., 2012; Liao et al., 2015). An in-depth study in tomato demonstrated that binding by α-kGDH E2 of SA acts upstream and affects the activity of the miETC, which helps to limit *tobacco mosaic virus* (*TMV*)-induced systemic viral inoculation (Liao et al., 2015). Similarly, the AOX pathway can also be induced by treatments with exogenous SA (Lee et al., 2011). Notably, the alternative pathway has also been demonstrated to be associated with disease resistance in plants. Evidence supporting this notion comes from some interesting findings—that overexpression of *AOX* in transgenic tomato and petunia significantly lowered the levels of *tomato spotted wilt virus* (*TSWV*) (Ma et al., 2011). Works on Cyt pathway inhibitors and salicylhydroxamic acid (SHAM) have further led to the proposition that the AOX pathway plays a key role in the resistance of tobacco plants to virus inoculation (Murphy et al., 1999; Ma et al., 2011).

Nevertheless, studies of the role of α*-KGDH* and *AOX* in biotic stress resistance are mainly limited to viruses, and it is still unclear in other kinds of pathogens. For example, tobacco resistance to TMV, *potato virus X* (*PVX*), and *cucumber mosaic virus* (*CMV*) can be stimulated by activation of the AOX pathway (e.g., antimycin A and potassium cyanide [KCN]) or repressed by inhibition of the AOX pathway by SHAM (Naylor et al., 1998; Mayers et al., 2005). In contrast, SHAM does not inhibit the induction of the pathogenesis-related protein 1 (*PR1*) or induction of resistance to another pathogen species *Eminia carotovora* by SA. Similarly, in *Arabidopsis*, SA-mediated resistance to *turnip crinkle virus* and SA-, or antimycin A-induced resistance to *turnip vein clearing virus*, as well as the induction of the *AOX* (the potential target for the chemicals), still remain active in *npr1* mutants (Kachroo et al., 2000; Wong et al., 2002). But the same mutation leads to loss of resistance to isolates of *Peronospora parasitica* and *Pseudomonas syringae* (Glazebrook et al., 1996; Rairdan and Delaney, 2002). These studies indicate that the signaling pathways for virus defense appear to branch below SA from that for fungi and bacteria, which involve *AOX* but are independent of *NPR1* (Murphy et al., 1999). However, *AOX* expression also responds strongly to bacterial inoculation (Cvetkovska and Vanlerberghe, 2012). Transgenic *AOX* knockdown *Nicotiana tabacum* plants inoculated with avirulent *P. syringae* pv. *maculicola* display a delayed reactive oxygen species (ROS) burst that manifests itself in a delayed hypersensitive response (HR) in an incompatible ETI response (Cvetkovska and Vanlerberghe, 2013). However, in another study of incompatible plant–pathogen interaction, transgenic tobacco (*Nicotiana attenuata*) plants silenced in the expression of *AOX* (irAOX) accumulate higher levels of ROS and HR after *P. syringae* pv. *tomato* DC3000 (*Pst*) inoculation (Zhang et al., 2012). These studies suggest that *AOX* is implicated during ETI response. But, to date, there has been little study to establish the role of *AOX*, as well as α*-KGDH E2* in the plant basal immunity.

Plant basal defense, which provides broad-spectrum defenses in compatible plant–pathogen interactions, is particularly important in crop cultivars. Tomato is one of the most economically important vegetable crops throughout the world, which often suffers from diseases and economic losses. In this study, we used virulent pathogens, *Pst*, as bacterial agents and explored the roles of mitochondrial α*-KGDH E2* and *AOX* in the plant basal defense. Our data indicate that α*-kGDH E2* in the TCA cycle, but not the miETC *AOX*, is involved in tomato basal defense against *Pst*. The defense functions of tomato α*-kGDH E2* are associated with lipoylation and SA signaling. This information helps to elucidate the mechanism(s) of mitochondria-involved plant defense.

## Materials and Methods

### Plant Growth and Chemical Treatment

The tomato (*Solanum lycopersicum*) lines used in most of the studies were mainly in the Zheza 809 wild-type background. The NahG transgenic line that cannot accumulate SA was in Money maker (MM) background and was obtained from the laboratory of J.D.G. Jones (Sainsbury Laboratory, Norwich, United Kingdom). Tomato plants were grown in trays and then transplanted to pots containing a growth substrate (peat:vermiculite, 3:1, vol/vol) in a plant growth room, under 12-h light/12-h dark photoperiod, 25°C/19°C day/night temperature, 500 μmol m^–2^ s^–1^ photosynthetic photon flux density, and 88% relative humidity. Tomato plants at the five-leaf stage were used for experiments. *Nicotiana benthamiana* plants were grown under similar conditions and used for transient overexpression assay after 6-week germination. For the chemical agent treatment, unless otherwise noted, 2 mM SA or water as control was sprayed onto plant leaves on both the adaxial and abaxial surfaces at 12 h prior to *Pst* inoculation.

### Virus-Induced Gene Silencing (VIGS) and Transient Overexpression

To generate virus-induced gene-silenced tomato plants, cDNA fragments of α*-kGDH E2* (380 bp) and *AOX1a* (594 bp) and *LIP2* (495 bp) were selected according to VIGS tool^1^. The polymerase chain reaction (PCR) fragments were inserted into pTRV2, and the primers used are shown in Supplementary Table S1. VIGS was performed by *Agrobacterium*-mediated transformation as previously described (Zhang et al., 2018). After viral inoculation, plants were maintained at 21°C before use in experiments.

α*-kGDH E2* and *AOX1a* gene fragments were amplified using primers shown in Supplementary Table S1 and cloned into pFGC5941 vector with an hemagglutinin (HA) tag and pAC402 vector with a green fluorescent protein (GFP) tag, respectively. After sequence confirmation, the plasmids were shuttled into *Agrobacterium tumefaciens* GV3101 strain. The transient overexpression in *N. benthamiana* was performed as previously described (Liao et al., 2015). Samples were collected 48 h later to assess expression by Western blot using an anti-HA and GFP antibody. The *A. tumefaciens*-infiltrated leaves were further used for pathogen inoculation experiments.

### Pathogen Inoculation and Disease Symptom Assays

Wild-type *Pst* was used for tomato plants to study the basal immunity, as there are no R proteins in Zheza 809 tomato. To avoid activation of the ETI response in *N. benthamiana*, *Pst hrcC*, which is deficient in the type III secretion system, was used for *N. benthamiana* inoculation. *Pst* or *Pst hrcC* were grown at 28°C in King’s B medium (10 g/L of peptone, 1.5 g/L of K_2_HPO_4_, 15 g/L of glycerol, and 5 g/L of MgSO_4_) with 50 mg/mL rifampicin. Bacterial cells were collected by centrifugation and dissolved in 10 mM MgCl_2_ to OD600 = 0.5. The bacterial inoculation of *Pst* was carried out by spraying the 10-time diluted bacterial suspension with 0.02% Silwet-L77 on the whole leaves of tomato plants. Leaves of *N. benthamiana* plants were infiltrated with *Pst hrcC* at the final concentration of OD600 = 0.01 using a syringe without a needle.

After pathogen inoculation, disease severity was assessed by the maximal quantum yield of PSII (*F*v/*F*m), trypan blue staining, or bacterial growth according to previously described methods (Zhang et al., 2015). The trypan blue staining was also quantified by ImageJ software based on the rate of dying area. As *A. tumefaciens*-carrying plasmids were infiltrated to *N. benthamiana* leaves for transient overexpression, which have common antibiotic resistance similar to bacterial *Pst* pathogens, the *Pst* bacterial growth was not measured to avoid potential confusion.

### SA Quantification

Endogenous SA content was measured in tomato leaves by HPLC-MS/MS (Agilent 6460; Agilent Technologies) with D4-SA (OlChemlm) as internal standards using the same method described previously (Zhang et al., 2018).

### RNA Isolation and RT-qPCR Assay

Total RNA was extracted from leaves using RNA simple Total RNA Kit (Tiangen, China), followed by DNase digestion, and reverse transcribed using a ReverTra Ace quantitative (qPCR) RT kit (Toyobo, Japan). Real time-quantitative PCR (RT-qPCR) assays were performed using SYBR Green PCR Master Mix Kit (Takara, Japan) on a Light Cycler 480 II detection system (Roche, Germany). *Actin2* was used as the appropriate reference gene. Gene-specific primers for RT-qPCR are listed in Supplementary Table S1.

### Respiration Analysis

Leaf respiration was assessed following the method of Millenaar et al. (2002) using a Clark-type oxygen electrode (Hansatech, King’s Lynn, United Kingdom). Total respiration was measured without any treatments. CN-resistant respiration and SHAM-resistant respiration were measured in the presence of 1 mM KCN and 3 mM SHAM, respectively.

### Diaminobenzidine Staining

Diaminobenzidine (DAB) staining was performed as previously described with minor changes (Xia et al., 2009). Leaf samples were vacuum infiltrated with 1 mg mL^–1^ DAB in 50 mM Tris-HCl (pH 3.8) and incubated overnight in dark at room temperature. Then, leaf samples were rinsed in 80% (vol/vol) ethanol for 10 min at 70°C, finally mounted in destaining solution (lactic acid/phenol/water, 1:1:1, vol/vol/vol). Pictures were taken under a microscope (Zeiss, Germany).

### Statistical Analysis

At least three independent biological replicates were performed for each determination. Unless otherwise stated, each biological replicate contained an independent sample that was pooled of two leaves, each from a different plant. The experiments were independently performed three times. The obtained data were subjected to analysis of variance using SAS software, version 8 (SAS Institute), and tested for significance using Tukey test at the 5% level.

## Results

### Effects of *Pst* Inoculation on Gene Transcripts, Mitochondrial Respiration, and SA Content

To examine the changes in the expression of genes involved in mitochondrial respiration, the transcript levels of 24 relevant genes were assayed at 8 and 12 h post inoculation with *Pst* (hpi). Among these, the SA biosynthesis- and signaling-related genes, including phenylalanine ammonia-lyase (*PAL2/4/6*), enhanced disease susceptibility 1 (*EDS1*), peptidyl arginine deiminase 4 (*PAD4*), *NPR1*, and *PR* family members, are up-regulated in response to *Pst* inoculation, which were also reported in a previous independent study (Yang et al., 2015). The transcripts of NAD-dependent isocitrate dehydrogenase (*ICDH*) in the TCA cycle and several miETC-related genes, including genes encoding succinate dehydrogenase of complex II (*SDH1*and *SDH2*), *AOX* family members (*AOX1a* and *AOX1b*), type II NAD(P)H dehydrogenases (*NDA1*and *NDB2*), and uncoupling proteins (*UCP1* and *UCP2*), were also significantly increased, especially at 12 hpi. By contrast, other seven genes clustered in another group were down-regulated in response to *Pst* inoculation, including TCA cycle-related genes α*-kGDH E2*, lipoyl synthase (*LIP1*), octanoyltransferase (*LIP2*) and mitochondrial β-ketoacyl-acyl carrier protein synthase (*mtKAS*), miETC-related genes cytochrome c oxidase subunit (*COX1*), and *AOX1c*, as well as SA biosynthesis-related gene isochorismate synthase (*ICS*).

**FIGURE 1:**
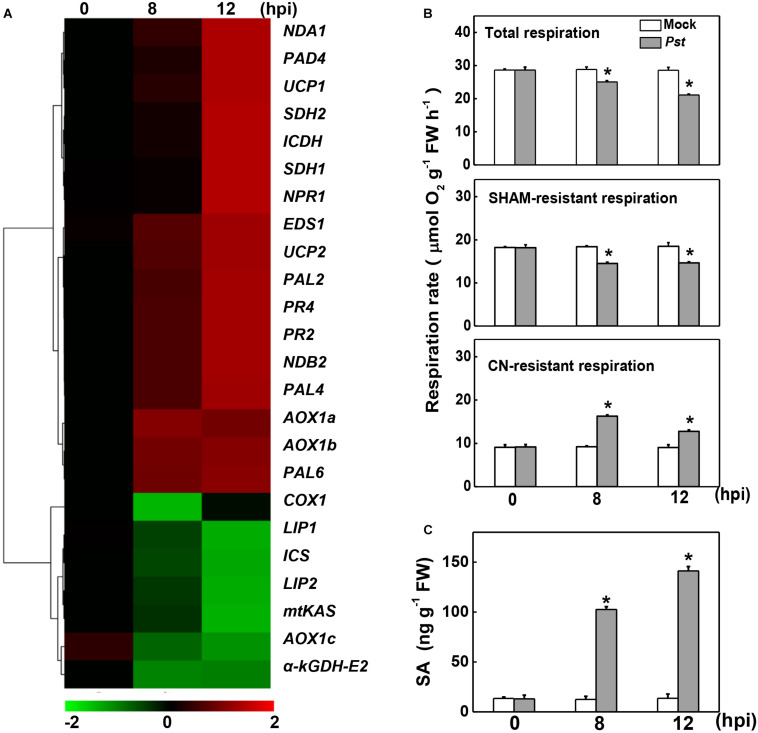
Effects of *Pseudomonas syringae* pv. *tomato* DC3000 (*Pst*) inoculation on gene expression, mitochondrial respiration, and salicylic acid (SA) content in tomato. **(A)** Changes in the gene transcripts 8- and 12-h post inoculation (hpi) with *Pst* based on cluster analysis. The intensity of the red or green color represents the degree of up- or down-regulation of the tested genes, respectively. The transcript levels of genes were determined by real-time quantitative PCR, with the *Actin2* gene serving as an internal control. **(B)** Effects of *Pst* inoculation on the rate of total respiration, salicylhydroxamic acid (SHAM)-resistant respiration and cyanide (CN)-resistant respiration at indicated time. **(C)** Effects of *Pst* inoculation on endogenous SA content in tomato leaves. The asterisk indicates a significant difference between treatments (*P* < 0.05, Tukey test). The results in panels **(B,C)** are presented as mean values ± SD; *n* = 3. The above experiments were repeated three times with similar results.

Changes in the rate of mitochondrial respiration and the endogenous SA content were further detected upon *Pst* inoculation. *Pst* inoculation caused a 12.58% decrease in total respiration rate at 8 hpi, followed by a significant decline at 12 hpi. A similar change occurred in the SHAM-resistant respiration rate, which decreased by 21.18% and 20.88% at 8 and 12 hpi, receptively. By contrast, *Pst* inoculation caused a 76.88% increase in the CN-resistant respiration rate at 8 hpi and a 41.39% increase at 12 hpi (Figure 1B). Meanwhile, consistent with previous studies on tomato (Yang et al., 2015; López-Gresa et al., 2017), the SA content was significantly induced and dramatically increased up to 10-fold at 12 hpi (Figure 1C).

### α*-kGDH E2*, but Not *AOX* Functions in Plant Basal Immune Response to *Pst*

**FIGURE 2:**
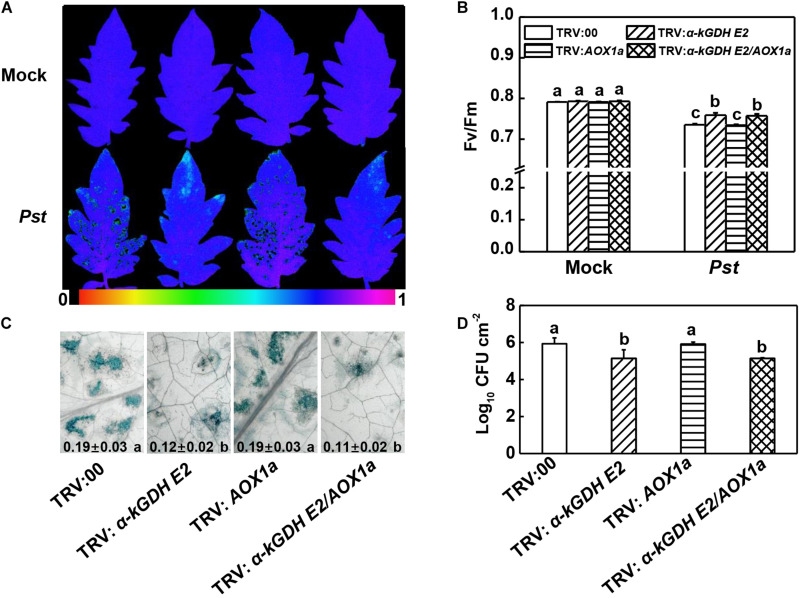
Effects of α*-kGDH E2* and *AOX1a* silencing on plant defense against *Pseudomonas syringae* pv. *tomato* DC3000 (*Pst*) inoculation in tomato. **(A)** Representative images of leaf maximum quantum yield of PSII (*F*v/*F*m) at 3 days post inoculation (dpi) with *Pst*. The color gradient scale at the bottom indicates the magnitude of the fluorescence signal represented by each color. **(B)** *F*v/*F*m values at 3 dpi. **(C)** Representative images for trypan blue staining of *Pst*-inoculated leaves at 3 dpi. Quantificative data are shown in each figure. **(D)** Bacterial growth at 3 dpi. The results in panels **(B–D)** are presented as mean values ± SD; *n* = 5. Different letters indicate significant a differences between treatments (*P* < 0.05, Tukey test). The above experiments were repeated three times with similar results.

To study the roles of tomato α*-kGDH E2* and *AOX* in the defense against *Pst*, we generated α*-kGDH E2-* and *AOX1a-*silenced plants via VIGS approach. RT-PCR was used to examine the efficiency of VIGS. Notably, under *Pst*-inoculated condition, the transcript levels of α*-kGDH E2* and *AOX1a* significantly decreased by 63.76% and 65.01%, in α*-kGDH E2-*silenced (TRV: α*-kGDH E2*) and *AOX1a*-silenced plants (TRV: *AOX1a*), respectively, as compared with the empty-vector control (TRV:00) (Supplementary Figures S1A,B). At 3 days post inoculation (dpi) with *Pst*, TRV: α*-kGDH E2* plants exhibited less reduction in the *F*v/*F*m and cell death, as well as increased *PR1*, *PR2*, and *PR4* transcripts at 12 hpi (Figures 2A–C and Supplementary Figure S1C). In contrast, *AOX1a*-silencing did not result in a significant difference compared with TRV:00 control. Also, α*-kGDH E2/AOX1a* gene cosilencing had similar *F*v/*F*m value, cell death quantification, and *PRs* transcripts level when compared with *a-kGDH E2* single gene-silenced plants. These parameters were corroborated with the proliferation of *Pst* in tomato leaves; bacterial growth in α*-kGDH E2-*silenced but not that of *AOX1a*-silenced leaves was significantly lower than that in the leaves of TRV:00 at 3 dpi (Figure 2D). Based on these findings, we assumed that the α*-kGDH E2* negatively regulated plant basal defense against bacterial pathogens.

**FIGURE 3:**
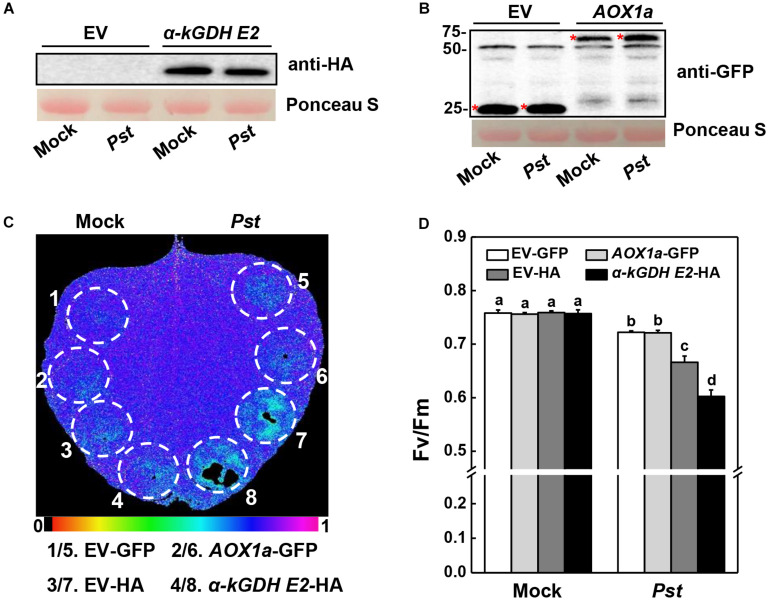
Effects of tomato gene α*-kGDH E2* and *AOX1a*-transient overexpression on plant defense against *Pseudomonas syringae* pv. *tomato* DC3000 *hrcC* (*Pst hrcC*) inoculation in *N. benthamiana* inoculation. **(A,B)** Tomato α*-kGDH E2* and *AOX1a* were transiently overexpressed in *N. benthamiana*, 48 h later, leaf samples were collected for proteins detection by Western blotting with an anti-HA antibody **(A)** and anti-GFP antibody **(B)**. Protein loading was verified by Ponceau S staining. **(C)** Representative image of gene overexpressed leaf maximum quantum yield of PSII (*F*v/*F*m) at 3 days post inoculation (dpi) with *Pst hrcC*. The color gradient scale at the bottom indicates the magnitude of the fluorescence signal represented by each color. **(D)** *F*v/*F*m values at 3 dpi. The results in panel **(D)** are presented as mean values ± SD; *n* = 5. Different letters indicate significant differences between treatments (*P* < 0.05, Tukey test). The above experiments were repeated three times with similar results.

Next, we constructed vectors carrying α*-kGDH E2* with an HA tag or *AOX1a* with a GFP tag and infiltrated them into *N. benthamiana* leaves for transient overexpression. Western blotting showed that α-kGDH E2 and AOX1a were abundantly expressed (Figures 3A,B). These leaves were then inoculated with *Pst hrcC*, which is deficient in the type III secretion system and can avoid triggering ETI response. Results in Figure 3 showed that overexpression of α*-kGDH E2* in *N. benthamiana* rendered more susceptibility to *Pst hrcC* inoculation, as reflected by significantly decreased *F*v/*F*m (Figures 3C,D). However, overexpression of *AOX1a* did not affect the defense against *Pst* inoculation. Taken together, these results indicate that tomato α*-kGDH E2* plays a negative role in plant basal immune response, but *AOX1a* seems to have no apparent function in the basal defense against *Pst*.

### Lipoylation of LIP2 to α-kGDH E2 Is Involved in Plant Basal Immunity Against *Pst*

**FIGURE 4:**
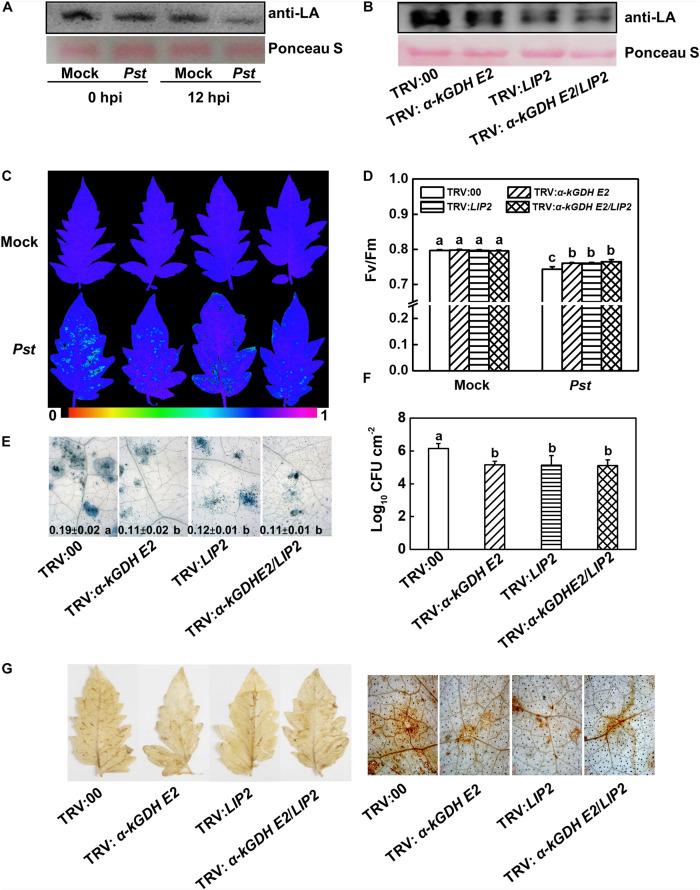
Effects of lipoylation of LIP2 to α-kGDH E2 on the plant defense against *Pseudomonas syringae* pv. *tomato* DC3000 (*Pst*) in tomato. **(A,B)** Effects of *Pst* inoculation and silencing *LIP2* on lipoylation of α*-kGDH E2*. **(A)** The lipoylation of α-kGDH E2 was detected by immunoblot analysis with an anti-lipoic acid (LA) antibody in tomato plants at 0 and 12 h post inoculation (hpi) with *Pst*. **(B)** Effects of silencing α*-kGDH E2* and *LIP2* on the lipoylation of α-kGDH E2 in tomato plants. Protein loading was verified by Ponceau S staining. **(C)** Representative images of leaf maximum quantum yield of PSII (*F*v/*F*m) at 3 days post inoculation (dpi) with *Pst*. The color gradient scale at the bottom indicates the magnitude of the fluorescence signal represented by each color. **(D)** *F*v/*F*m values at 3 dpi. **(E)** Representative images for trypan blue staining of *Pst*-inoculated leaves at 3 dpi. Quantificative data are shown in each figure. **(F)** Bacterial growth at 3 dpi. **(G)** Hydrogen peroxide (H_2_O_2_) contents at 24 hpi. The results in panels **(D–F)** are presented as mean values ± SD; *n* = 5. Different letters indicate significant differences between treatments (*P* < 0.05, Tukey test). The above experiments were repeated three times with similar results.

The lipoylation of α-kGDH E2 is essential for its catalytic activity, and α-kGDH E2 was previously reported to be lipoylated by LIP2 (Ewald et al., 2014). Therefore, changes in the lipoylation of α-kGDH E2 in response to *Pst* inoculation were examined, which showed that the lipoylation level significantly decreased in response to *Pst* at 12 hpi (Figure 4A). We then generated *LIP*2- and *LIP2/*α*-kGDH E2-* cosilenced plants (Supplementary Figures S2A,B). Silencing of *LIP2* in tomato plants did result in a noticeable reduction in the lipoylation (Figure 4B).

We compared plant basal defense between α*-kGDH E2-* and *LIP2-*silenced plants. Based on the data of *F*v/*F*m, cell death, bacterial growth, and *PRs* gene expression (*PR1*, *PR2*, and *PR4*), silencing *LIP2* reduced plant susceptibility and increased the *PRs* transcripts to a similar level of α*-kGDH E2-*silenced plants. Notably, cosilencing of these two genes did not result in a further change in plant defense (Figures 4C–F and Supplementary Figure S2C), suggesting that α*-kGDH E2* and *LIP2* work in the same pathway in the basal defense response.

We then analyzed the accumulation of hydrogen peroxide (H_2_O_2_) via DAB staining in the empty-vector control and target gene-silenced leaves at 24 hpi. Silencing of α*-kGDH E2*, *LIP2*, or gene cosilencing dramatically suppressed the accumulation of H_2_O_2_ to a similar level (Figure 4G). Overall, these results suggest that lipoylation of LIP2 to α-kGDH E2 plays a negative role in plant basal immunity.

### α*-kGDH E2* Acts in Downstream of SA Defense Pathway Against *Pst* Inoculation

**FIGURE 5:**
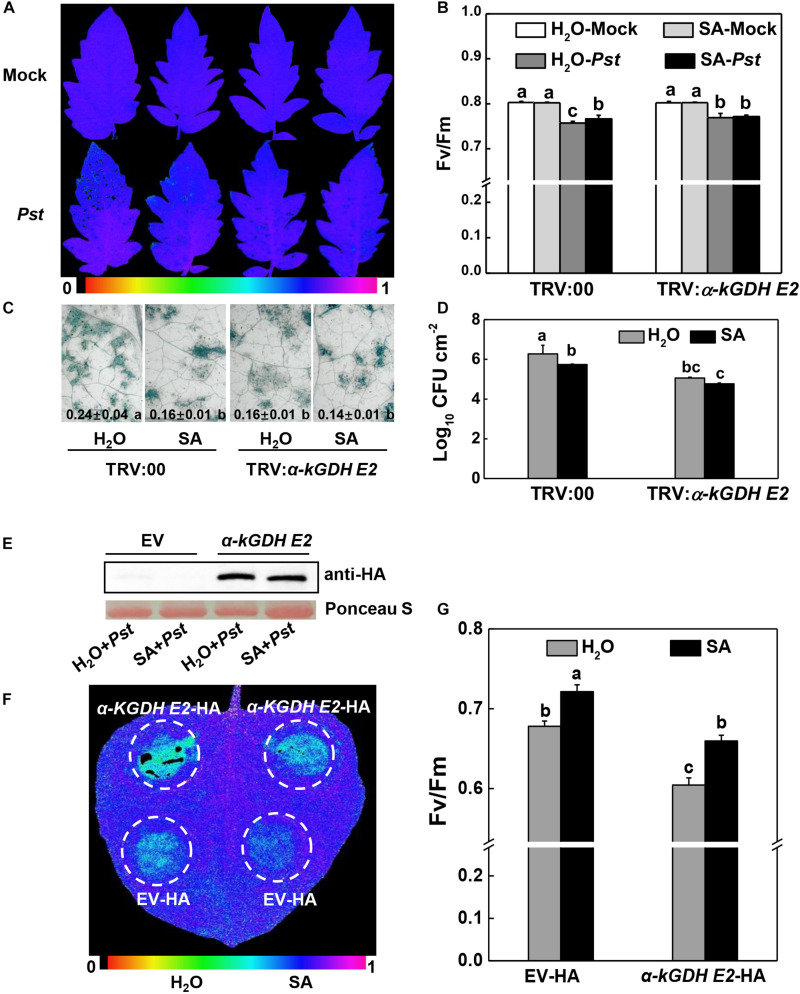
Effects of SA application on plant defense against *Pseudomonas syringae* pv. *tomato* DC3000 (*Pst*) inoculation in α*-kGDH E2-*silenced tomato or transiently overexpressed *N. benthamiana* plants. **(A–D)** Effects of SA application on the resistance to *Pst* in α*-kGDH E2*-silenced tomato plants. 2 mM SA or water as control was sprayed onto tomato leaves at 12 h prior to *Pst* inoculation. **(A)** Representative images of leaf maximum quantum yield of PSII (*F*v/*F*m) at 3 days post inoculation (dpi) with *Pst*. The color gradient scale at the bottom indicates the magnitude of the fluorescence signal represented by each color. **(B)** *F*v/*F*m values at 3 dpi. **(C)** Representative images for trypan blue staining of *Pst*-inoculated leaves at 3 dpi. Quantificative data are shown in each figure. **(D)** Bacterial growth at 3 dpi. **(E–G)** Effects of SA application on the resistance to *Pst hrcC* in α*-kGDH E2* transient overexpression *N. benthamiana* plants. **(E)** Tomato α*-kGDH E2* was transiently overexpressed in *N. benthamiana*; 48 h later, leaf samples were collected for proteins detection by Western blotting with an anti-HA antibody. Protein loading was verified by Ponceau S staining. 2 mM SA or water as control was sprayed onto leaves at 12 h prior to *Pst hrcC* inoculation. **(F)** Representative image of leaf *F*v/*F*m at 3 dpi. The color gradient scale at the bottom indicates the magnitude of the fluorescence signal represented by each color. **(G)** *F*v/*F*m values at 3 dpi. The data are shown in panels **(B–D,G)** as means ± SD (*n* = 5), and different letters indicate significant differences between treatments (*P* < 0.05, Tukey test). The above experiments were repeated three times with similar results.

Tomato α-kGDH E2 has been reported to be an SABP (Liao et al., 2015). We thus determined the relationship between α*-kGDH E2* and SA in the basal defense against *Pst*. In the TRV:00 control plants, the *Pst* inoculation-induced PSII damage and bacterial growth were significantly alleviated by pretreatment with exogenous SA. Silencing of α*-kGDH E2* again diminished *Pst* inoculation-induced PSII damage or cell death, but they were not further reduced by exogenous SA pretreatment (Figures 5A–D). Similarly, exogenous SA pretreatment did not lead to further induction of *PR1* gene transcripts in *a-kGDH E2-*silenced plants (Supplementary Figure S3).

The failure of SA to improve defense in α*-kGDH E2-*silenced plants might be attributable to the already lower α*-kGDH E2* gene expression in these plants. We therefore tested the role of exogenous SA in the α*-kGDH E2* overexpressed *N. benthamiana* plants. Strikingly, *Pst*-caused damage of the *F*v/*F*m was evidently attenuated by the exogenous SA pretreatment in α*-kGDH E2-*overexpressed leave*s* (Figures 5E–G).

**FIGURE 6:**
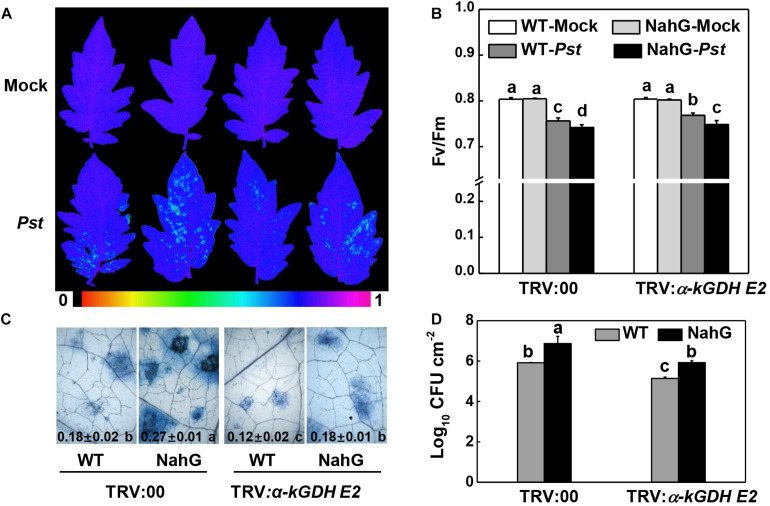
Effects of silencing α*-kGDH E2* on plant defense against *Pseudomonas syringae* pv. *tomato* DC3000 (*Pst*) inoculation in SA accumulation- defective-transgenic NahG and wild-type tomato plants. **(A)** Representative images of leaf maximum quantum yield of PSII (*F*v/*F*m) at 3 days post inoculation (dpi) with *Pst*. The color gradient scale at the bottom indicates the magnitude of the fluorescence signal represented by each color. **(B)** *F*v/*F*m values at 3 dpi. **(C)** Representative images for trypan blue staining of *Pst*-inoculated leaves at 3 dpi. Quantitative data are shown in each figure. **(D)** Bacterial growth at 3 dpi. The results in panels **(B–D)** are presented as mean values ± SD; *n* = 5. Different letters indicate significant differences between treatments (*P* < 0.05, Tukey test). The above experiments were repeated twice with similar results.

We asked whether α*-kGDH E2* functions in the defense response of NahG plants that are impaired in SA accumulation. The results showed that NahG plants were more susceptible to *Pst* inoculation compared with control WT plants, whereas silencing α*-kGDH E2* gene led to higher defense against *Pst* in both WT and NahG backgrounds (Figure 6).

## Discussion

Mitochondrial respiration is an important metabolic process in plant innate immunity (Colombatti et al., 2014). Particularly, α*-KGDH E2* of the TCA cycle and AOX pathway of miETC participate in plant defense, mostly against viral pathogens in plants. In this study, we showed that tomato α*-kGDH E2* but not *AOX* negatively regulated plant basal defense to bacterial pathogen *Pst* in an SA-dependent process.

In this study, the SHAM-resistant Cyt pathway respiration and expression of α*-kGDH E2* were repressed, whereas the CN-resistant AOX pathway was significantly induced by the *Pst* inoculation (Figure 1). These results are in accordance with previous reports in which application of elicitor harpin (virulence factors produced by bacterial pathogens such as *P. syringae*) inhibited mitochondrial respiration and causing a strong induction of *AOX* in *Arabidopsis* cells (Krause and Durner, 2004). Several studies have described *AOX1a* as the most stress-responsive *AOX* gene, whereas *AOX1c* expression is relatively stable or even decreased in response to stresses or elicitor treatment (Clifton et al., 2006; Czobor et al., 2019).

VIGS and transient overexpression approaches were used to further understand the biological functions of α*-kGDH E2* and *AOX* in the defense against virulent *Pst*. We found that the susceptibility to *Pst* was significantly reduced by silencing α*-kGDH E2* in tomato plants, but increased by overexpressing α*-kGDH E2* in *N. benthamiana* plants. In contrast, silencing or overexpressing of *AOX1a* gene did not have significant effects as compared with that of the control counterpart (Figures 2, 3). To the best of our knowledge, this is the first evidence that α*-kGDH E2* acts as a negative regulator in plant defense against bacterial pathogen *Pst*. Previously, α*-kGDH E2* was reported to function in the plant defense against TMV through modulating the miETC AOX pathway and the associated mitochondrial oxidative phosphorylation in tomato (Liao et al., 2015). However, in contrast to the plant defense to viral pathogens, the tomato defense against *Pst* is independent on AOX pathway as evidenced by both gene silencing and overexpression experiments (Figures 2, 3). Thus, the function of *AOX* in plant defense seems to be pathogen species-specific. For instance, *N. attenuata AOX* contributes to resistance to piercing-sucking insects but not to *Manduca sexta* larvae (Zhang et al., 2012). In the incompatible plant–bacterial pathogen interaction, *AOX* knockdown *N. tabacum* displayed a delayed ROS and HR in response to *P. syringae* pv. *Maculicola* (Cvetkovska and Vanlerberghe, 2013), but showed higher levels of ROS and HR after *Pst* inoculation (Zhang et al., 2012). The present study indicates that α*-kGDH E2*, but not *AOX*, is a key component of basal immunity, which warrants further investigation in broader compatible plant–bacteria pathogen systems.

In the TCA cycle, the lipoylation of α-kGDH E2 is essential for its catalytic activity, and it was reported to be lipoylated by LIP2 in *Arabidopsis* (Ewald et al., 2014). The deficiency in the lipoylation of α-kGDH E2 and pyruvate dehydrogenase leads to an early onset of fatal lactic acidosis in humans (Tort et al., 2014). Whether the lipoylation of LIP2 to α-kGDH E2 functions in plant basal immunity remains unclear. In this study, the transcripts of *LIP2* greatly decreased in response to *Pst* inoculation (Figure 1A). Also, *Pst* inoculation caused a significant decrease in the lipoylation of α-kGDH E2 (Figure 4B). Most importantly, silencing *LIP2* contributed to an enhanced resistance to *Pst*, as well as higher level of SA-dependent marker gene *PR1*, *PR2*, and *PR4* to the same level with α*-kGDH E2-*silenced plants (Figure 4 and Supplementary Figure S2), indicating that the lipoylation of LIP2 to α-kGDH E2 plays a negative role in plant defense against *Pst*. Excessive accumulation of ROS causes severe oxidative damage to plants, and control of oxidative damage is essential for plants to survive under and recover from stresses (Samsatly et al., 2018). Previous reports have suggested that α*-kGDH* is not only a target of ROS but also could significantly contribute to the control of ROS accumulation in the mitochondria (Tretter and Adam-Vizi, 2005). In this study, silencing α*-kGDH E2* or *LIP2* both significantly reduced the accumulation of H_2_O_2_ (Figure 4G). Therefore, these results suggest that silencing α*-kGDH E2* or *LIP2* may alleviate the H_2_O_2_ accumulation to prevent the plant from oxidative damage during *Pst* inoculation. However, we cannot exclude a possibility that α*-kGDH E2* in plants is associated with physiological responses such as nutritional shift and carbon partitioning (Araújo et al., 2012), which affect pathogen growth as well. In addition, 2-oxoglutarate (2-OG), a key organic acid of the TCA cycle, is an obligatory substrate for α-kGDH (Bailey and Nathan, 2018). Previous evidence has indicated that 2-OG plays an important role in the metabolism of glucosinolate, flavonoid, and alkaloid (Araújo et al., 2014), which are widely distributed secondary metabolites with different biological functions in plants, including the defense against pathogen inoculation (Hunziker et al., 2020; Nabavi et al., 2020). Thus, it is tempting to speculate that the down-regulation of α*-kGDH E2* and *LIP2* may also induce the accumulation of 2-OG, which may play a role in basal immune responses.

Salicylic acid-dependent signaling controls the activation of complex plant defense responses to combat microbial pathogens (Zhang and Li, 2019). In agreement with previous studies (Yang et al., 2015; López-Gresa et al., 2017), the SA content dramatically increased in response to *Pst* inoculation, which was accompanied by a significant increase in the transcripts of SA biosynthesis- and signaling-related genes (Figure 1). Tomato α-kGDH E2 was proven to be SABP, and α*-kGDH E2* acted as a negative regulator of SA-dependent defense to TMV (Liao et al., 2015). In this study, SA treatment and silencing α*-kGDH E2* both increased resistance to *Pst*. SA did not further enhance defense against *Pst* in α*-kGDH E2-*silenced tomato plants, but did reduce the susceptibility in *N. benthamiana* plants transiently overexpressing α*-kGDH E2* (Figure 5). Additionally, silencing α*-kGDH E2* gene led to higher defense against *Pst* in both WT or NahG backgrounds (Figure 6). Thus, tomato α*-kGDH E2* was proposed to function as a negative regulator of SA-dependent defense against *Pst*. By the way, the *AtICS* is the most important gene for SA biosynthesis in *Arabidopsis* upon pathogen inoculation (Garcion et al., 2008). Unlike that in *Arabidopsis*, in this study, the *PAL* expression but not that of *ICS* was induced by *Pst* inoculation (Figure 1). The expression profile of *ICS* was in agreement with previous genome-wide studies in tomato–*Pst* interactions (Yang et al., 2015). Similarly, in a study on pepper plants, *Obuda pepper virus* inoculation markedly induced the expression of three *PAL* genes, whereas that of *ICS* gene was not modified (Dziurka et al., 2016). Also, multiple pathogen infections with virulent or avirulent strains of the *Pst* pathogens on soybean were associated with suppression of *ICS* gene expression (Shine et al., 2016). Thus, pathogen-induced SA biosynthesis may use different pathways in different species, which need further studies.

In summary, the study reveals a novel function of a mitochondrial TCA cycle enzyme α-kGDH E2, but not miETC AOX, in the orchestration of plant basal immunity in a compatible plant–pathogen interaction. The study not only provides new insights into the function of mitochondrial respiration against bacterial pathogens, but also identifies new targets and markers for the development of improved cultivars that are better equipped to combat pathogens.

## Data Availability Statement

The raw data supporting the conclusions of this article will be made available by the authors, without undue reservation.

## Author Contributions

KS designed the experiments. QM and YL performed most of experiments and analyzed the data. HF, PW, and WZ assisted in experiments and discussed the results. QM and KS wrote the manuscript. KS and GA edited the manuscript. All authors contributed to the article and approved the submitted version.

## Conflict of Interest

The authors declare that the research was conducted in the absence of any commercial or financial relationships that could be construed as a potential conflict of interest.

## Footnotes

1 https://vigs.solgenomics.net/

## Supplementary Material

Supplementary Figure 1 Real-time RT-PCR analysis of α*-kGDH E2*, *AOX1a* and *PR genes* expression in virus-induced genes silencing (VIGS) tomato plants as infected by *Pseudomonas syringae* pv. *tomato* DC3000 (*Pst*). **(A,B)** The efficiency of VIGS was examined in α*-kGDH E2-* **(A)** and *AOX1a-* **(B)** silenced tomato plants. Samples were collected at 12 hours post inoculation (hpi) with *Pst*. **(C)** The transcript expression of *PR1*, *PR2* and *PR4* at 0 and 12 hpi. The *Actin2* gene was used as the internal control. The control sample was normalized to 1. Data are presented as mean values ± SD; *n* = 3. Different letters indicate a significant differences between treatments (*P* < 0.05, Tukey’s test).

Supplementary Figure 2 Real-time RT-PCR analysis of α*-kGDH E2*, *AOX1a* and *PR genes* expression in virus-induced genes silencing (VIGS) tomato plants as infected by *Pseudomonas syringae* pv. *tomato* DC3000 (*Pst*). **(A,B)** The efficiency of VIGS was examined in α*-kGDH E2-* **(A)** and *LIP2-* **(B)** silenced tomato plants. Samples were collected at 12 hours post inoculation (hpi) with *Pst*. **(C)** The transcript expression of *PR1*, *PR2* and *PR4* at 12 hpi. The *Actin2* gene was used as the internal control. The control sample was normalized to 1. Data are presented as mean values ± SD; *n* = 3. Different letters indicate a significant differences between treatments (*P* < 0.05, Tukey’s test).

Supplementary Figure 3 Real-time RT-PCR analysis of *PR1* expression in empty-vector control and α*-kGDH E2*-silenced tomato plants with exogenous SA application as infected by *Pseudomonas syringae* pv. *tomato* DC3000 (*Pst*). The *Actin2* gene was used as the internal control. The control sample was normalized to 1. Data are presented as mean values ± SD; *n* = 3. Different letters indicate a significant differences between treatments (*P* < 0.05, Tukey’s test).
