# Supplementary material for: An Essential Role of Mitochondrial α-Ketoglutarate Dehydrogenase E2 in the Basal Immune Response Against Bacterial Pathogens in Tomato
Source: Front Plant Sci. 2020 Oct 30;11:579772. doi: 10.3389/fpls.2020.579772 (PMC7661389; doi:10.3389/fpls.2020.579772)
Supplement: Supplementary Figure 1 — Real-time RT-PCR analysis of α-kGDH E2, AOX1a and PR genes expression in virus-induced genes silencing (VIGS) tomato plants as infected by Pseudomonas syringae pv. tomato DC3000 (Pst). (A,B) The efficiency of VIGS was examined in α-kGDH E2- (A) and AOX1a- (B) silenced tomato plants. Samples were collected at 12 hours post inoculation (hpi) with Pst. (C) The transcript expression of PR1, PR2 and PR4 at 0 and 12 hpi. The Actin2 gene was used as the internal control. The control sample was normalized to 1. Data are presented as mean values ± SD; n = 3. Different letters indicate a significant differences between treatments (P < 0.05, Tukey’s test). [file Data_Sheet_1.pdf]

Supplementary Material

Figure S1

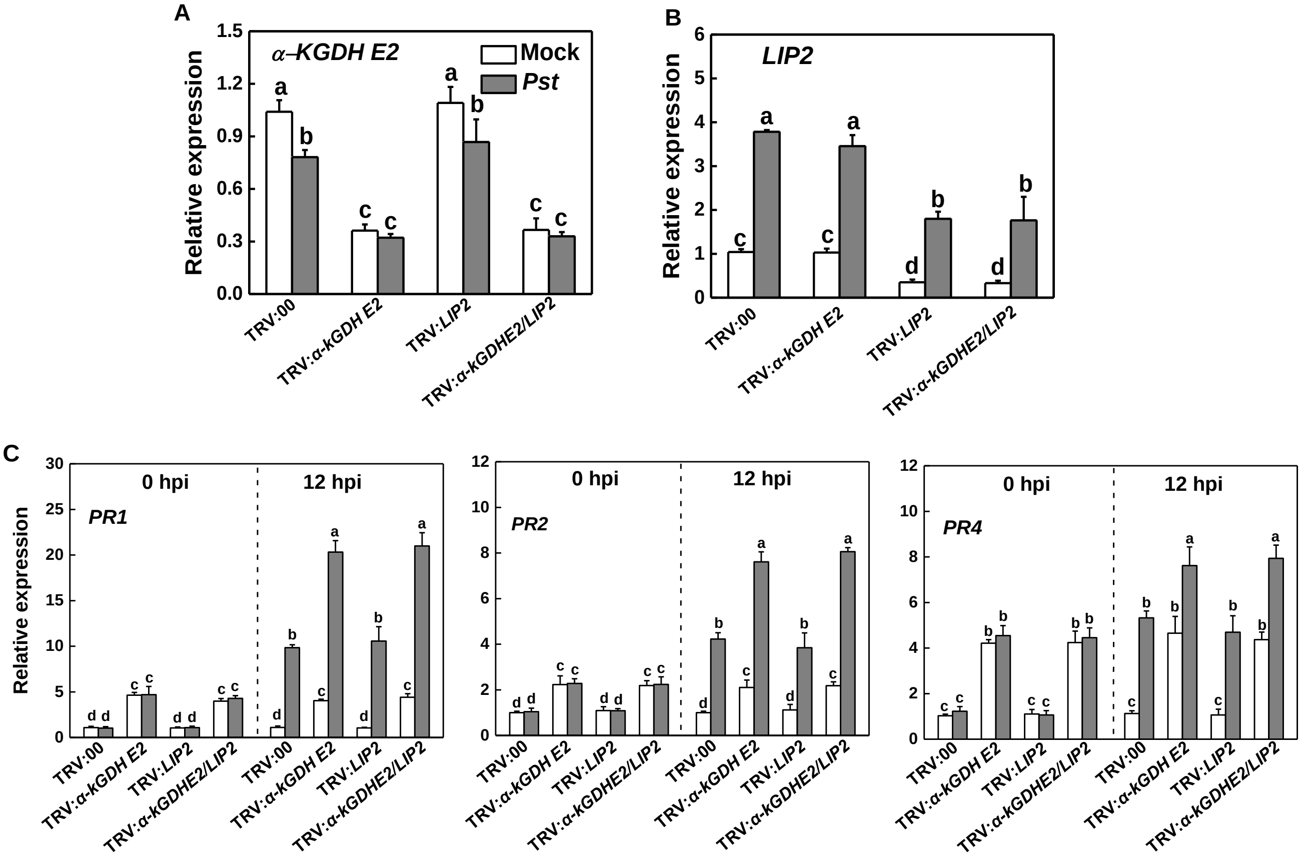

Figure S1. Real-time RT-PCR analysis of *α-kGDH E2, AOX1a* and *PR genes* expression in virus-induced genes silencing (VIGS) tomato plants as infected by *Pseudomonas syringae* pv. *tomato* DC3000 (*Pst*).

(A-B) The efficiency of VIGS were examined in *α-kGDH E2-* (A) and *AOX1a-* (B) silenced tomato plants. Samples were collected at 12 hours post inoculation (hpi) with *Pst*. (C) The transcript expression of *PR1*, *PR2* and *PR4* at 0 and 12 hpi. The *Actin2* gene was used as the internal control. The control sample was normalized to 1. Data are presented as mean values ± SD; $n = 3$. Different letters indicate significant differences between treatments ($P<0.05$, Tukey's test). The above experiments were repeated three times with similar results.

Figure S2

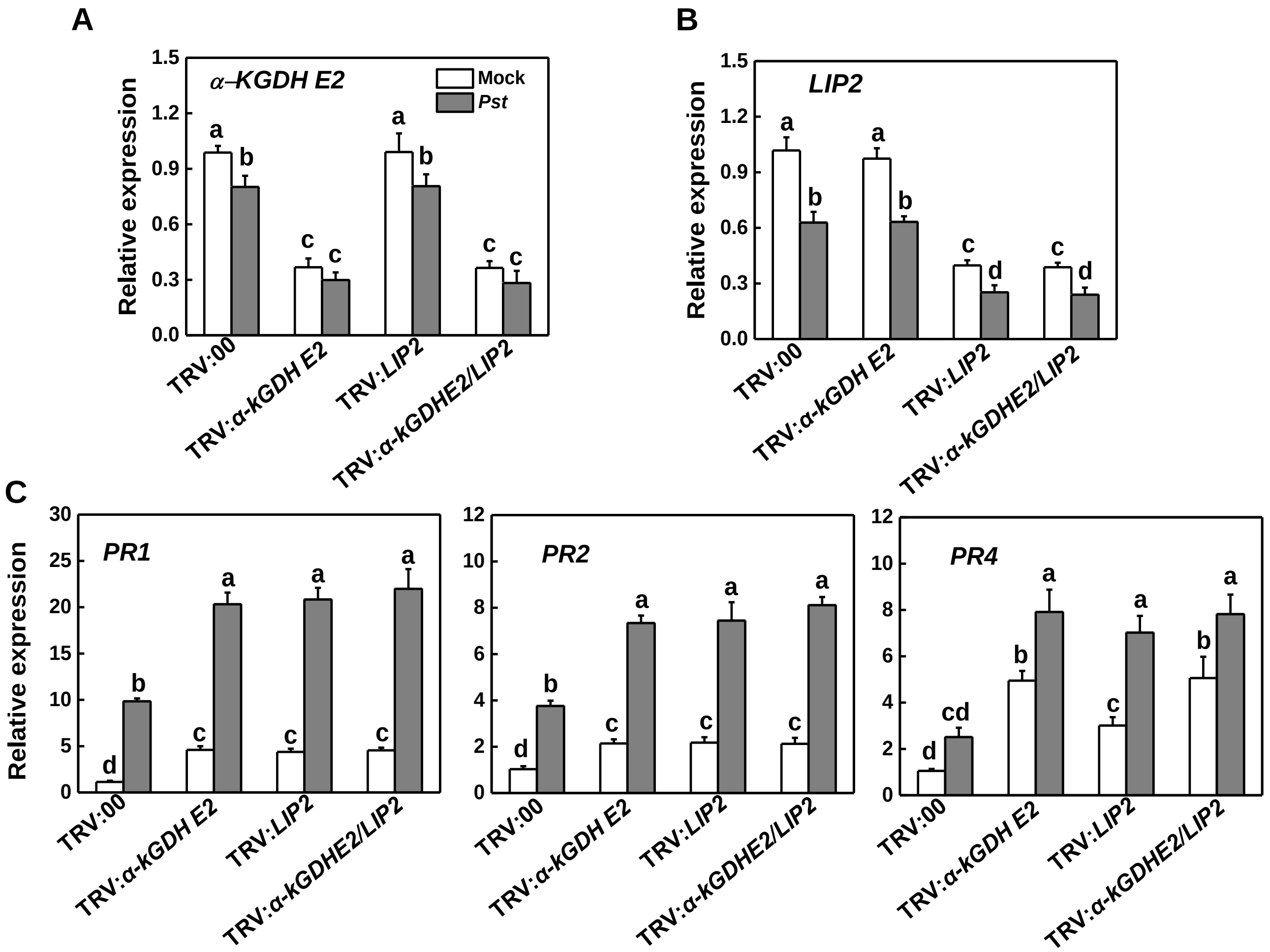

Figure S2. Real-time RT-PCR analysis of *α-kGDH E2, AOX1a* and *PR genes* expression in virus-induced genes silencing (VIGS) tomato plants as infected by *Pseudomonas syringae* pv. *tomato* DC3000 (*Pst*).

(A-B) The efficiency of VIGS were examined in *α-kGDH E2*- (A) and *LIP2*- (B) silenced tomato plants. Samples were collected at 12 hours post inoculation (hpi) with *Pst*. (C) The transcript expression of *PR1*, *PR2* and *PR4* at 12 hpi. The *Actin2* gene was used as the internal control. The control sample was normalized to 1. Data are presented as mean values ± SD; *n* = 3. Different letters indicate significant differences between treatments ($P<0.05$, Tukey's test). The above experiments were repeated three times with similar results.

Figure S3

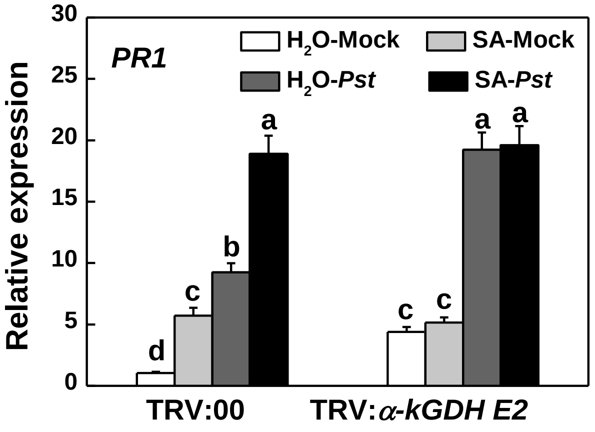

Figure S3. Real-time RT-PCR analysis of *PR1* expression in empty-vector control and *α-kGDH E2*-silenced tomato plants with exogenous SA application as infected by *Pseudomonas syringae* pv. *tomato* DC3000 (*Pst*).

The *Actin2* gene was used as the internal control. The control sample was normalized to 1. Data are presented as mean values ± SD; $n = 3$. Different letters indicate significant differences between treatments ($P<0.05$, Tukey's test). The above experiments were repeated three times with similar results.

Table S1. Primers used in this study

Gene	Accession No.	Primer pairs	Assay
SlPR4	Solyc00g174340.1	F: 5’-TAGTCTGGCGCAACTCAGTC-3’<br>R: 5’-TGCAAGAAATGAACCACCAT-3’	qPCR
SlPR2	Solyc01g106620.1	F: 5’- CTCGGTACGTCTTGGTTGTG -3’<br>R: 5’- TCCAGTTACCTGGTGGATCA -3’	qPCR
SlPAD4	Solyc02g032850.2	F: 5’-TCCACGTTGTGTTGGATTCT-3’<br>R: 5’-TGACAACCATCCCATTATCG-3’	qPCR
SlLIP2	Solyc02g077460.1	F: 5’-GGTGTTACGTCTTTGAAGAATGA-3’<br>R: 5’-AGCGATCAACTGCTCCTGTA-3’	qPCR
		F: 5’-GCtctagaTTACCTGGAGGCACTGA-3’<br>R: 5’-CGCggatccATCCCACAAGGCACAA-3’	pTRV2 (for VIGS)
SlNDA1	Solyc02g078900.2	F: 5’-TGAATGGTTGCGAGTTCC-3’<br>R: 5’-GTTTCCCAATGTCGGTGA-3’	qPCR
SlNDB2	Solyc02g079170.3	F: 5’-GGAATGGCTGTCTGGTCT-3’<br>R: 5’-CCACTCATCGGTTGCTAA-3’	qPCR
SlSDH1	Solyc02g085350.2	F: 5’-TTGCATGGTTGGACAAGATT-3’<br>R: 5’-ATGTCTCTTGTGTGCGGAAG-3’	qPCR
SlSDH2	Solyc02g093680.2	F: 5’-AGCGATAGGGCTAAGTTGGA-3’<br>R: 5’-TATCCATGATCCATCGGTTG-3’	qPCR
SlPAL2	Solyc05g056170.2	F: 5’-AATCGCTATGGCCTCGTACT-3’<br>R: 5’-TGTCAACTGCCTCCTCTGTC-3’	qPCR
SlICS	Solyc06g071030	F: 5'-GGCTTTAGCTGGAACACGG-3'<br>R: 5'-CAATCTTCTTCTTATGCACTC CC-3'	qPCR
SlEDS1	Solyc06g071280.2	F: 5’-TAGAAACAGCTGCAAATGGC-3’<br>R: 5’-AGATGCCTGTTTGTGCTGAG-3’	qPCR
SlNPR1	Solyc07g040690.2	F: 5’-TTTCGACTTCTTCGCTGATG-3’<br>R: 5’-TCCAGCTTCGTGTTGCTATC-3’	qPCR
SlmtKAS	Solyc07g049570.2	F: 5’-GACGAGCAGTTGTGGCTAAA-3’<br>R: 5’-GGGCATCCAATTAGCATCTT-3’	qPCR
SlLIP1	Solyc07g054540.2	F: 5’-CCTGATCAAGGAAGTGGTCA-3’<br>R: 5’-CCCAGGATCACCTCTGAAAT-3’	qPCR

SlAOX1c	Solyc08g005550.2	F: 5’-TAAGGCGATTCGAGCACA-3’<br>R: 5’-CCATTAAGACAAGTCCACGT-3’	qPCR
SlAOX1a	Solyc08g075540.2	F: 5’-TCATTACCAAGGACAACAGC-3’<br>R: 5’-GGAACAAAATAGTGACGGAC-3’	qPCR
		F: 5’-TGCtctagaAAGCCAAATGTATACGAACG-3’<br>R: 5’-CCGgaattcGATATAATTTCATCAAGAAT-3’	pTRV2 (for VIGS)
		F: 5’-CCGctcgagATGATGACCCGTGGAGCAAC-3’<br>R: 5’-CGCggatccGTGATACCCAAGTGGTGCTG-3’	pAC402 (for over-expression with GFP tag)
SlAOX1b	Solyc08g075550.2	F: 5’-TCCTCCACTGTAAATCCC-3’<br>R: 5’-AAATACGCCTTGAACTGC-3’	qPCR
SlICDH	Solyc08g077920.2	F: 5’-GCACCTGATATTGCTGGAAA-3’<br>R: 5’-CTTGGATGCGATCAGCTTTA-3’	qPCR
SlPR1	Solyc09g007010.1	F: 5’-CTCAAGAGCGGGTGATTGTA-3’<br>R: 5’-ATAGTCTGGCCTCTCGGACA-3’	qPCR
SlPAL4	Solyc09g007890.1	F:5’-AATCGCGATGGCTTCTTACT-3’<br>R:5’-CCCAACGAATTCACATCTTG-3’	qPCR
SlUCP1	Solyc09g011920.2	F: 5’-GCACTTACAACTGGTGCGTT-3’<br>R: 5’-AGCTCCAGAATAACGCCTTG-3’	qPCR
SlPAL6	Solyc10g086180.1	F: 5’-GCCCAATTCTCAGAGCTTGT-3’<br>R: 5’-CTTGGATTCCTTCCTGCTGT-3’	qPCR
SlUCP2	Solyc11g010500.1	F: 5’-CGGTTGCTG TTG GTGTTAAG-3’<br>R: 5’-AACCCATCCTGGTGGTAGAG-3’	qPCR
SlCOX1	Solyc11g063610.1	F: 5’-ATTGGTCTGTTCCGATTC-3’<br>R: 5’-TGCCGCTACCCACTTCTA-3’	qPCR
Slα-kGDH-E2	Solyc12g005080.1	F: 5’-AATGATGTACATTGCGCTGAC-3’<br>R: 5’-GGCGTGGATCTTCTACCAC-3’	qPCR
		F: 5’-CGCggattcCAAAGAACGGGAAAGACGAG-3’<br>R: 5’-CCGgaatccTTGTCTTTTCTATCTCAGCA-3’	pTRV2 (for VIGS)
		F: 5’-GCGctcgagATGGGCGAATCCATAAGCGATG GCAC-3’<br>R: 5’-GCGtctagaTCAAGCGTAATCTGGACCATCG TATGGG TAAACATCAAGGAGTAGGCGGCGTG-3’	pFGC1008 (for over-expression with HA tag)
SlACTIN2	Solyc03g078400.2	F: 5’-TGGTCGGAATGGGACAGAAG -3’<br>R: 5’-CTCAGTCAGGAGAACAGGGT -3’	qPCR
